# Contrasting nitrogen-use strategies and photosynthetic carbon-water trade-offs in *Pinus tabulaeformis* plantations under different forest management modes

**DOI:** 10.3389/fpls.2026.1891622

**Published:** 2026-08-27

**Authors:** Lianjin Zhang, Yanbo Hu, Fan Mo, Yang Yu, Jiajun Chen, Zeyu Zhou, Manyi Du, Huanying Feng, Ziyuan Zhou, Di Liu

**Affiliations:** 1 Experimental Center of Forestry in North China, National Permanent Scientific Research Base for Warm Temperate Zone Forestry of Jiulong Mountain in Beijing, Chinese Academy of Forestry, Beijing, China; 2 State Key Laboratory of Efficient Production of Forest Resources, Key Laboratory of Tree Breeding and Cultivation of National Forestry and Grassland Administration, Research Institute of Forestry, Chinese Academy of Forestry, Beijing, China; 3 Northwest Institute of Investigation and Planning, National Forestry and Grassland Administration, Xi`an, China; 4 Central South Academy of Inventory and Planning, National Forestry and Grassland Administration, Changsha, China

**Keywords:** forest management modes, leaf functional traits, photosynthetic characteristics, *Pinus tabulaeformis*, randomized forest management

## Abstract

**Introduction:**

Forest management practices can alter canopy light environments and competitive intensities, thereby reshaping leaf functional traits and photosynthetic physiology. However, how different management modes coordinate leaf morphology, carbon gain, water use, and nitrogen utilization in coniferous plantations remains unclear.

**Methods:**

A field experiment was conducted in a *Pinus tabulaeformis* plantation under four management modes: structure-based forest management (SM), randomized forest management (RM), close-to-nature forest management (NM), and an unmanaged control (CK). Leaf morphological traits, gas exchange parameters, carbon and nitrogen biogeochemical traits, and nitrogen use efficiency were analyzed and compared.

**Results:**

All modes significantly decreased leaf water content (LWC), with the largest reduction (8.44%) under SM. Only SM significantly reduced specific leaf area (SLA) by 27.3%. Net photosynthetic rate (Pn), stomatal conductance (Gs), and transpiration rate (Tr) were significantly increased by management, particularly under SM and RM. RM achieved the highest Pn (+59% vs. CK), whereas SM increased Gs and Tr the most (2.38 and 2.07 times CK). All treatments decreased instantaneous water use efficiency (WUE) and stomatal limitation (Ls). SM and RM significantly increased total nitrogen (TN) but decreased C/N. Contrasting nitrogen-use patterns emerged: SM increased leaf nitrogen content per unit area (Na) to 1.55 times CK and elevated δ¹^5^N by 16.08%, suggesting nitrogen accumulation; RM increased photosynthetic nitrogen use efficiency (PNUE) by 28% without markedly increasing Na, suggesting more efficient nitrogen utilization. Correlation analysis further indicated that Pn was significantly positively correlated with TN and negatively with C/N, while Na was positively correlated with Pn, Gs, Tr, and δ¹³C, but negatively with LWC and SLA.

**Discussion:**

Different forest management modes exhibited contrasting nitrogen-use patterns and altered the carbon-water trade-off. RM achieved the highest photosynthetic rate and PNUE while avoiding excessive nitrogen accumulation, thereby appearing to strike a better balance between carbon gain and nitrogen economy. Therefore, RM appears promising for enhancing carbon fixation and nitrogen use efficiency in *P*. *tabulaeformis* plantations, though multi-year, multi-site validation is needed. This study provides early-stage mechanistic insights into trait-based forest management and highlights the importance of integrating carbon–water–nitrogen synergies, while acknowledging the need for further biochemical validation and broader spatial-temporal replication.

## 1 Introduction

Plant functional traits (PFTs) typically refer to relatively stable and measurable morphological, physiological, and phenological characteristics that directly or indirectly influence plant growth, reproduction, and survival (He et al., 2018). These traits often appear in coordinated, functional packages known as plant trait syndromes, which embody plant adaptive strategies to their environment and are closely linked to biophysical and biogeochemical processes, as well as ecosystem functions, making them crucial for assessing biodiversity and ecosystem processes (Díaz, 2025; Ma et al., 2023; Wang H. et al., 2022). PFTs serve as a bridge between plants and the environment, enabling them to respond to and influence environmental conditions (Song et al., 2023). Extensive research indicates that nearly all PFTs exhibit significant variation across broad environmental gradients, serving as effective indicators for predicting changes in ecosystem function under environmental shifts (He et al., 2023). Currently, research on PFTs has gradually shifted from focusing on single functional traits to examining multiple functional traits and trait networks (Li et al., 2024; Wright et al., 2004). They have been widely applied in ecological restoration and management, biodiversity and ecosystem functions, predicting productivity, responses to environmental change, and community assembly (Chen et al., 2023; Wang et al., 2023; Zheng et al., 2023).

Leaves serve as the core organs for photosynthesis, respiration, and transpiration in plants. Their traits, such as specific leaf area, leaf thickness, and nutrient content, provide insights into how plants acquire, utilize resources, and survive in their environments. Leaf functional traits (LFTs) reflect plant adaptation strategies to varied environments. They are central to understanding plant economic strategies, particularly through the Leaf Economics Spectrum (LES), which describes a universal trade-off between rapid resource acquisition and long-term resource conservation (Wright et al., 2004). Plants with high SLA tend to have thin, lightweight leaves, allowing for rapid carbon gain and higher mass-based photosynthetic rates, supporting a “live fast, die young” approach with short leaf lifespans (Ali et al., 2016; Zhao et al., 2025). Conversely, plants with low SLA invest in tougher, more durable leaves, which are long-lived and indicative of a conservative strategy suited for nutrient-poor or harsh environments (Zhao et al., 2025).

Structure-based forest management (SM), randomized forest management (RM) and close-to-nature forest management (NM) are three forest management methods that have been widely applied in China in recent years (Hui et al., 2019; Wan et al., 2023). SM assumes that the system structure determines function and aims to cultivate a healthy, stable, high-quality, high-efficiency forest; it uses structural parameters to guide adjustments that optimize forest structure (Hui, 2020; Liao et al., 2025). RM focuses on adjusting the distribution pattern of forests; its theoretical basis is that a random structure unit has incomplete and unbalanced resource allocation, which can reduce symmetric competitive pressure. Its management purpose is to promote more stable productivity (Hui et al., 2021; Xu et al., 2021). NM focuses on biodiversity, resilience, and climate adaptation in managed forests and forested landscapes; its basic idea is to use the natural succession process of forests to balance the ecological, social, and economic functions of forests. It aims to promote positive forest succession (components, structures, and processes characteristic of natural forests and cultural woodlands) and shorten the natural succession time of forests, thereby improving the conservation values and climate resilience of multifunctional forests (Larsen et al., 2022; Renčo et al., 2024; Roitsch et al., 2023). These three forest management methods have been applied to improve the quality of plantations and are feasible ways to achieve multi-objective management of plantations (Wan et al., 2022). As forest management is shifting from timber-oriented to multifunctional objectives, understanding how management affects plant physiological strategies becomes increasingly important.

Forest management practices represent systematic human interventions that can directly and profoundly alter stand structure, light conditions, moisture, nutrient availability, carbon sequestration, and biodiversity, thereby influencing tree survival strategies and growth (Fan et al., 2024; Neudam et al., 2023; Tinya et al., 2023; Wang et al., 2024; Wang Z. et al., 2022). Previous studies have also indicated that leaf functional traits of *P*. *tabulaeformis* are influenced by various forest management practices including water and nutrient management (nitrogen addition, nitrogen deposition, and soil water stress) (Song and Hou, 2020; Cui et al., 2023; Jiang et al., 2015; Guo et al., 2017; Wang et al., 2011), tending (artificial tending and gap-model thinning) (Li et al., 2014; Zhao and Bao, 2018), and light environments (shading) (Zheng et al., 2013). In summary, current research primarily focuses on the effects of natural factors, site conditions, and thinning on specific aspects of needle functional traits. However, systematic empirical studies and mechanistic explanations regarding how different management regimes specifically regulate these traits remain lacking. This gap prevents us from understanding the ecological effects of management practices at the level of needle functional traits.

The Chinese pine (*Pinus tabulaeformis* Carr.) is an endemic dominant species of warm temperate forests in China that grows mainly in northern China (Wu and Feng, 1994). It is one of China’s main afforestation coniferous species, occupies a very important position in mountain vegetation restoration, and plays important roles in wood production, carbon sequestration, and ecological services. It grows on an area of more than 2.5 million ha, with an estimated stocking volume of 0.13 billion m^3^ (National Forestry and Grassland Administration of China, 2019). Previous research has revealed significant intraspecific variation in leaf functional traits of *P*. *tabulaeformis*, influenced by multiple factors including geographic patterns (latitude and longitude) (Lei et al., 2025; Zhang et al., 2017), environmental gradients (elevation and precipitation) (Liu et al., 2021; Zhang et al., 2023), disturbance factors (such as fire) (Gu et al., 2022), developmental stages (forest age and leaf age) (Wang Y. et al., 2022; Wen et al., 2024), and urban environments (Su et al., 2021). This substantial variation in response to environmental gradients, disturbance regimes, and stand development stages makes *P*. *tabulaeformis* an ideal species to investigate the physiological responses to forest management interventions.

Few studies have examined the accompanying changes in leaf functional traits after management in *P*. *tabulaeformis* forests. Changes in leaf functional traits under the three forest management methods are unclear. Different management practices induce structural and environmental changes in *P*. *tabulaeformis* forests, thereby reshaping the relationship between stands and their surroundings. Therefore, it is essential to link microscopic leaf trait responses with macroscopic management measures to elucidate physiological and ecological adaptation mechanisms of trees under human-induced management practices and enrich the theoretical framework of management ecology. Based on the above considerations, we proposed the following hypotheses: (1) forest management would modify leaf morphological traits (e.g., decrease SLA and LWC) and enhance photosynthetic rates; (2) different management modes would induce contrasting nitrogen-use strategies, with SM promoting nitrogen accumulation (higher Na and δ¹^5^N) and RM promoting nitrogen utilization (higher PNUE); (3) there would be fundamental correlations between carbon gain and water loss across all management modes, reflected in decreased instantaneous WUE. This study aims to elucidate the mechanisms by which different forest management practices affect leaf functional traits of *P*. *tabulaeformis* by quantitatively analyzing differences in leaf morphological traits, gas exchange parameters, carbon and nitrogen biogeochemical traits, and nitrogen use efficiency. The findings are of great significance for optimizing forest management measures and synergistically enhancing stand productivity and ecological stability of *P*. *tabulaeformis* forests, providing a scientific basis for precision sustainable management and improvement of *P*. *tabulaeformis* plantations.

## 2 Materials and methods

### 2.1 Study site description

**Figure 1:**
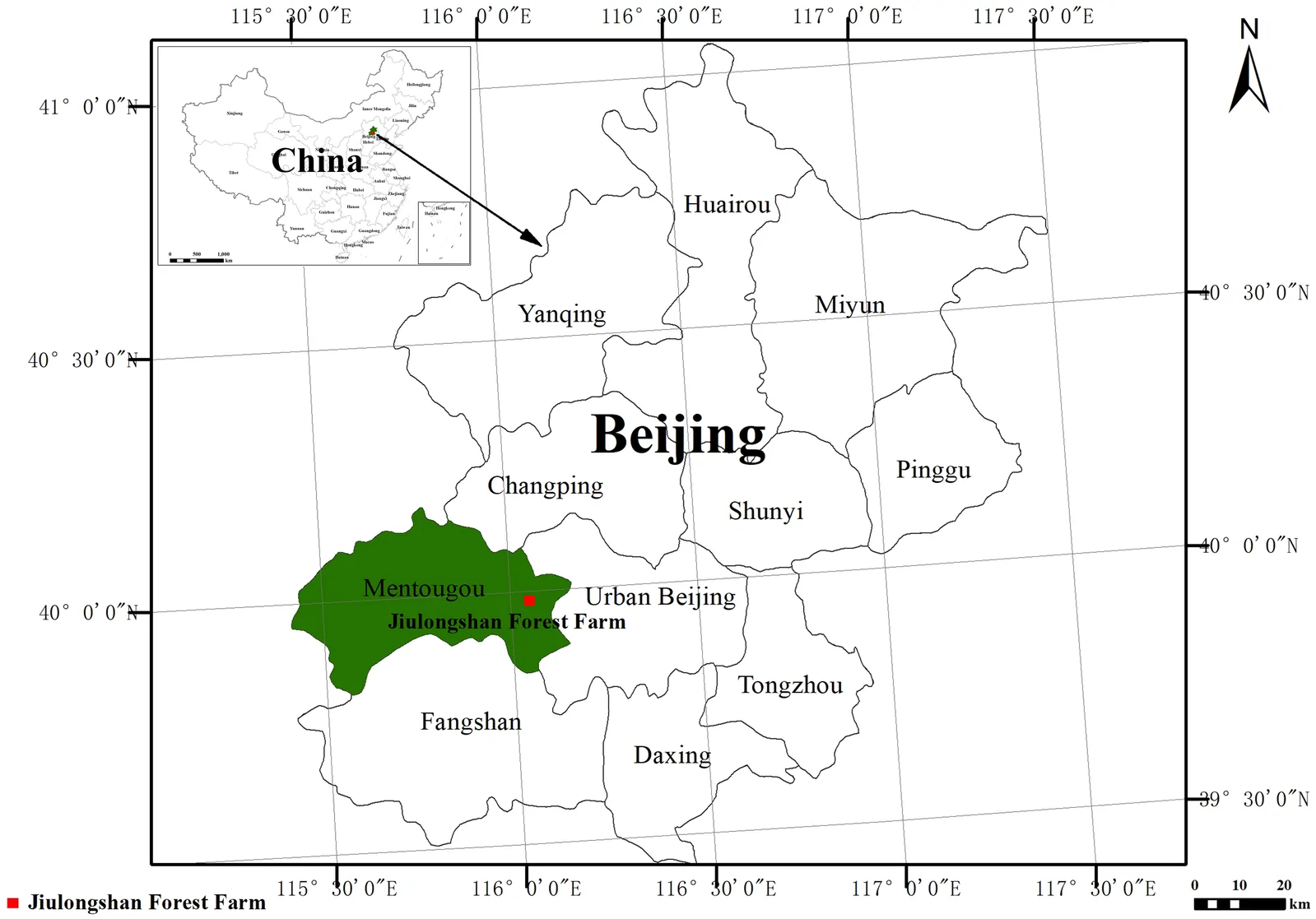
The study plots, located in Jiulongshan Forest Farm, Mentougou District, Beijing, PR China.

This research was carried out at the Jiulong Mountain, part of the Experimental Center of Forestry in North China under the Chinese Academy of Forestry. The site is situated to the east of the Taihang Mountains, near Beijing (115°59′–116°07′E, 39°54′–39°59′N), with elevations ranging from 100 to 997 m above sea level (**Figure 1**). The area experiences a temperate continental climate influenced by monsoon patterns. Mean annual temperature is 11.8 °C, while average yearly precipitation and total evaporation are approximately 630 mm and 1870 mm, respectively. The soil is classified as brown rocky mountainous forest soil, characterized by high stone content and shallow layers. Vegetation consists mainly of warm-temperate coniferous and broadleaf plantations, with dominant species primarily composed of *P*. *tabulaeformis*, *Platycladus orientalis*, and *Quercus variabilis* (Zhang et al., 2018, 2022).

### 2.2 Experimental design

In the spring of 2019, 12 permanent plots (30 m×30 m each) were established in *P*. *tabulaeformis* plantations at Jiulongshan Forest Farm. The location and site conditions of these plots were nearly identical. The stand is 54 years old and situated on a north-facing middle slope at approximately 733 m a.s.l. It has an average slope of 22°, a soil depth of about 45 cm, a litter layer thickness of about 6 cm, and a canopy closure of 0.80. All trees with diameter at breast height (DBH) ≥5 cm in each plot were positioned with a Topcon GTS602 (Topcon, Tokyo, Japan) autofocus total station. Tree species, DBH, height, and crown diameter were recorded. To avoid edge effects, a 3-meter buffer zone was established inside each plot boundary. The 12 permanent sample plots were allocated to the four management treatments using a completely stochastic optimization design method (Zhang and Hui, 2015; Wan et al., 2019), which can ensure that the initial state of this test is basically consistent among the four management treatments. The initial state includes the key management parameters (Uniform angle index (*W_i_*), Mingling (*M_i_*), Dominance (*U_i_*), and Crowding (*C_i_*)), stand density, average DBH, average height, average crown width, and basal area in each management treatment. The basic stand characteristics of the plots are presented in **Table 1**. One way ANOVA indicated no significant differences in characteristics (e.g. stand density, average DBH, average height, average crown width, and basal area) among the four treatments (p > 0.05) (**Table 1**), which confirmed that the plots were well matched across treatments prior to management intervention.

**Table 1:** Basic stand characteristics of the plots.

Management modes	Tree species composition	Stand density (trees.hm^-2^)	Average DBH (cm)	Average height (m)	Average crown width (m)	Basal area (m^2^.hm^-2^)
SM	9 *P. tabulaeformis* 1 other broadleaf trees	1055 ± 40a	19.92 ± 0.50a	11.33 ± 0.28a	3.83 ± 0.26a	32.82 ± 0.86a
RM	9 *P. tabulaeformis* 1 other broadleaf trees	1058 ± 5a	19.60 ± 0.49a	11.22 ± 0.03a	3.98 ± 0.19a	31.92 ± 1.43a
NM	9 *P. tabulaeformis* 1 other broadleaf trees	941 ± 83a	19.64 ± 0.43a	10.57 ± 0.65a	4.29 ± 0.29a	28.19 ± 1.24a
CK	9 *P. tabulaeformis* 1 other broadleaf trees	989 ± 62a	19.18 ± 0.64a	10.42 ± 0.37a	4.02 ± 0.16a	28.40 ± 0.48a

SM, RM, NM, and CK represent structure-based forest management, randomized forest management, close-to-nature forest management, and the control, respectively. Data are shown as means ± SE (n = 3). Values with different letters indicate significant effects at *p*< 0.05. Other broadleaf trees refer to *Syringa reticulata* var. amurensis, *Carya cathayensis*, and *Fraxinus chinensis*.

In the winter of 2019, SM, RM, and NM were conducted in the plots. Plots that were not subject to forestry operations served as controls (CK). SM primarily uses spatial structure parameters based on the spatial relationships of the four nearest-neighbor trees (Uniform angle index (*W_i_*), Mingling (*M_i_*), Dominance (*U_i_*), and Crowding (*C_i_*)) to guide adjustments to forest spatial structure. RM mainly involves creating more random structures through randomization. Randomization is the process of transforming the spatial distribution pattern of the target tree and its four nearest neighbors into a random structure (*W_i_* = 0.50). NM chiefly determines target tree density and harvests trees that hinder the growth of the target trees or significantly affect the health of the stand. For this study, the number of target trees was set at 150 per hectare; extrapolated by area, this equates to 13–14 trees per sample plot (900 m²). All treatments were applied consistently across the three replicate plots per treatment. All harvesting operations were confined exclusively to *P. tabulaeformis*, with no other tree species felled. To prevent soil compaction, ground disturbance, and damage to the forest floor, no wheeled or tracked machinery entered the plots at any stage. Felling was carried out entirely by hand-held chainsaws operated by skilled crews, and all resulting slash—including branches and tops—was manually collected and removed from the site to eliminate potential breeding grounds for pests and pathogens. Understory vegetation was not intentionally modified, and any incidental damage during felling and extraction was minimized through careful directional felling and manual handling. And the tree density intensities for SM, RM, and NM were 17.30%, 17.65%, and 17.95%, respectively. The corresponding volume harvesting intensities were 10.31%, 10.22%, and 10.48%, respectively. For a more detailed explanation of the three management treatments (SM, RM, and NM), please see the Attachment S1.

### 2.3 Samplings and measurements

In August 2024, three trees with average DBH that were healthy and in good growth were selected from each plot. Three branches per tree were harvested by using high pruning shears from the upper and middle canopy facing southeast. In each plot, the mean value measured from three branches and three trees was averaged to constitute one plot-level replicate; for each management mode, there were three independent plots (n = 3 per treatment), with each plot representing one replicate. All statistical analyses were performed on plot-level averages.

Gas exchange parameters of current-year needles, including net photosynthetic rate (Pn), intercellular CO_2_ concentration (Ci), stomatal conductance (Gs), and transpiration rate (Tr), were measured using a portable photosynthesis system Li-6400 (Li-Cor, USA) on the collected branches. Measurements employed red-blue light sources. Atmospheric CO_2_ concentration (Ca) entering the leaf chamber was controlled at 400 μmol·mol^-1^ to match current ambient levels—a standard condition in photosynthetic gas-exchange measurements that ensures comparability across studies and reflects realistic growth environments (Fa et al., 2025; Gao et al., 2014). Air temperature and relative humidity in the chamber were manually adjusted to 25°C and 60%, respectively, which were similar to ambient conditions. Photosynthetically active radiation (PAR) was set to 1000 μmol·m^-^²·s^-^¹, which is at or slightly above the light saturation point for *P. tabulaeformis* (Zhang et al., 2007), thereby allowing measurement of light-saturated net photosynthetic rates and capturing the maximum photosynthetic capacity of the needles (Fa et al., 2025; Gao et al., 2014). Each needle was allowed 5–10 min to equilibrate to chamber conditions until readings were stable and the coefficient of variation was< 1%. Stomatal conductance values were logged every 5s until stability was reached.

Instantaneous water use efficiency (WUE) was calculated by using Pn and Tr, and stomatal limitation (Ls) was calculated by using Ci and Ca. The formulas (Equations 1, 2) were as follows:

(1) WUE = Pn/Tr

(2) Ls=(Ci/Ca)×100%

Immediately after gas exchange measurements, all current-year needles were collected from three trees and branches per plot and bulked to one composite sample. Current-year needles were chosen for this study because they are the most physiologically active and responsive to immediate environmental changes. A total of 12 composite samples were obtained. Each composite sample was divided into two subsamples.

One subsample was weighed for fresh weight (FW). It was then scanned using a scanner to obtain needle images, and the needle leaf area (LA) was determined. This portion of needles was dried in an oven at 65°C until constant weight was achieved. The constant weight was recorded as dry weight (DW). SLA and leaf water content (LWC) (Equations 3, 4) were calculated as follows:

(3) LWC = (FW/DW)×100%

(4) SLA = LA/DW

The other subsample was also dried in an oven at 65°C until constant weight was achieved. After pulverization and sieving, it was sealed for storage and subsequently used to determine the general chemical properties: δ¹³C, δ¹^5^N, total carbon (TC), and total nitrogen (TN). TC and TN were quantified using an elemental analyzer. δ¹³C and δ¹^5^N were determined by coupling an elemental analyzer with an isotope ratio mass spectrometer. Nitrogen content in unit leaf (Na) and photosynthetic nitrogen use efficiency (PNUE) (Equations 5, 6) were calculated as follows:

(5) Na=100×TN/SLA

(6) PNEU=10×Pn/Na=Pn×SLA/(100×TN)

### 2.4 Statistical analysis

Data analysis and graphics were conducted in R software (ver. 4.1.2; R Development Core Team, Vienna, Austria). Differences in morphological traits, gas exchange parameters, carbon and nitrogen biogeochemical traits, and nitrogen use efficiency among management modes (SM, RM, NM, and CK) were tested using one-way analysis of variance (ANOVA) followed by Tukey’s honest significant difference (HSD) test for *post-hoc* multiple comparisons. All *post-hoc* comparisons were performed using Tukey’s HSD test to control for Type I error. Pearson correlation coefficients were used to calculate the correlations among morphological traits, gas exchange parameters, carbon and nitrogen biogeochemical traits, and nitrogen use efficiency. Prior to ANOVA, normality was assessed using the Shapiro–Wilk test (W > 0.90 for all variables) and homogeneity of variances using Levene’s test (p > 0.05 for all variables), confirming that no transformation was required. All data conformed to normal distribution and met the homogeneity of variance assumption. Statistical significance was set as *p*< 0.05. Figures were prepared using the ggplot2 package (Wickham, 2016).

## 3 Results

### 3.1 Morphological traits under different management modes

Forest management modes significantly affected the LWC and SLA. Both LWC and SLA were lower in forest management treatments than in CK (**Table 2**). Compared with CK, all forest management modes significantly decreased LWC (*p*< 0.05), with levels following the order: SM< NM< RM. SM significantly reduced SLA (*p*< 0.05), while the other two management modes only decreased SLA non-significantly (**Table 2**).

**Table 2:** Morphological traits in current-year needles under different management modes.

Management modes	SM	RM	NM	CK
LWC (%)	56.00 ± 0.12d	59.58 ± 0.61b	58.17 ± 0.71c	61.16 ± 0.91a
S LA (cm ^2^ .g ^-1^)	101.18 ± 6.86b	130.27 ± 16.06a	115.25 ± 5.81ab	139.16 ± 16.02a

LWC and SLA represent the leaf water content and specific leaf area, respectively. Data are presented as mean ± SE (n=3). Different lowercase letters within each row indicate significant differences among management modes (*p*< 0.05).

### 3.2 Gas exchange parameters under different management modes

**Figure 2:**
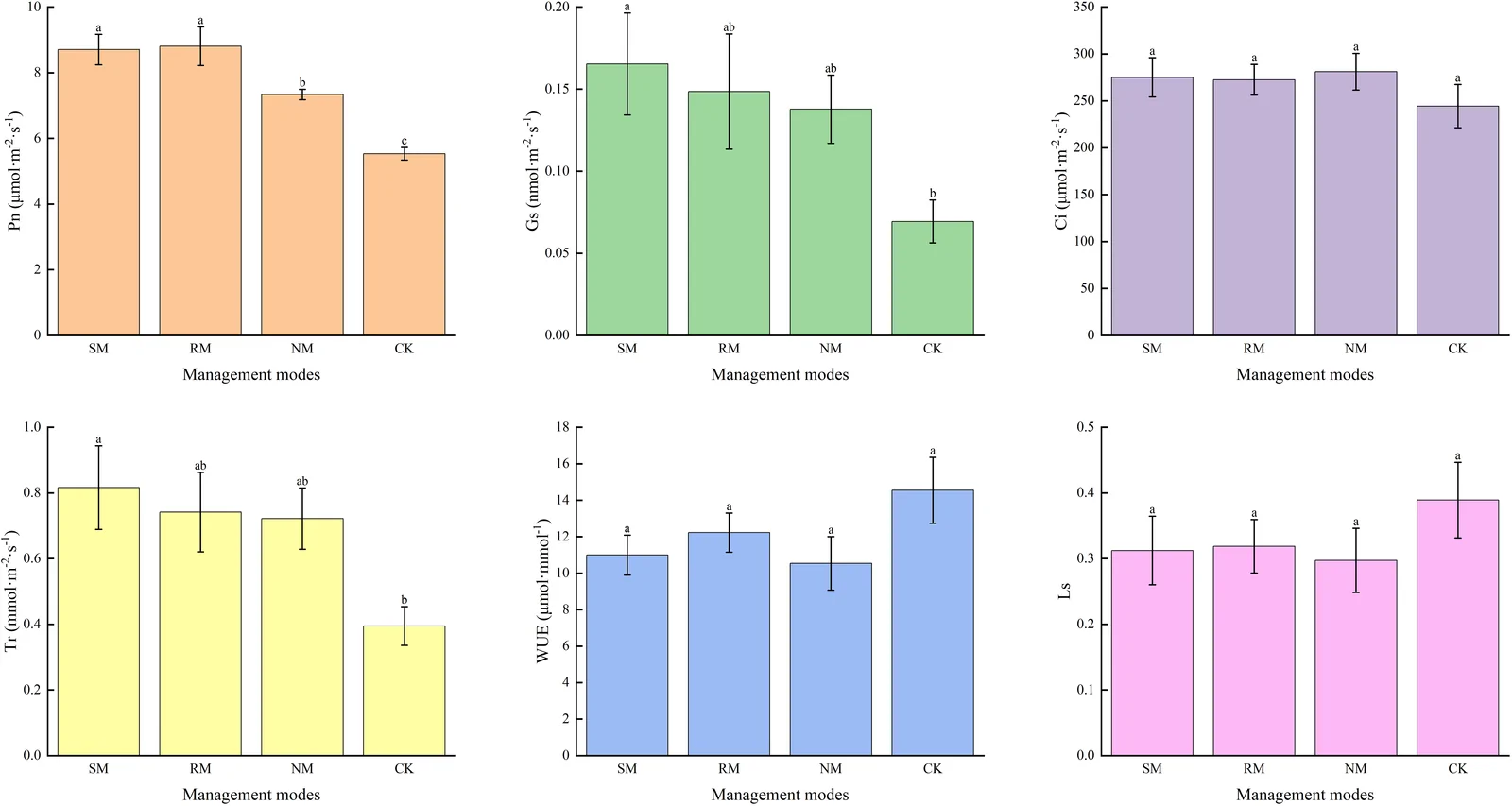
Gas exchange parameters under different management modes. Pn, Gs, Ci, Tr, WUE and Ls represent the net photosynthetic rate, stomatal conductance, intercellular CO_2_ concentration, transpiration rate, water use efficiency and stomatal limitation, respectively. Different lowercase letters indicate significant differences among management modes (*p*< 0.05). Error bars indicate standard deviation (n = 3).

Changes in gas exchange parameters under each management mode are shown in **Figure 2**.

Pn was effectively promoted by forest management modes and was significantly higher than that of the control (*p*< 0.05, **Figure 2**). Pn values of SM, RM, and NM were 1.57, 1.59, and 1.33 times that of CK, respectively. RM showed the highest Pn, which was significantly different from those of NM and CK (*p*< 0.05). RM also had a higher Pn than SM, but the difference was not significant.

Compared with CK, Gs and Tr were increased by forest management modes (**Figure 2**). Significant differences in Gs and Tr were found between SM and CK (*p*< 0.05), but not among the management modes. Gs and Tr of SM were 2.38 and 2.07 times those of CK, respectively. Although Gs and Tr in RM and NM were higher than those of CK, no significant differences were found between them (**Figure 2**).

Management modes had a certain impact on the Ci, WUE and Ls. Ci ranged from 244.38 to 281.09 μmol·mol^-^¹, and all three management modes increased the Ci by 11.51% to 15.02%, in the order RM>SM>NM (**Figure 2**). However, no significant difference was found among management modes or between them and CK. In contrast, management practices decreased WUE and Ls relative to CK, although these differences were not statistically significant (**Figure 2**). WUE varied from 10.54 to 14.54 μmol·mmol^-^¹; SM, RM, and NM decreased it by approximately 24.42%, 15.96% and 27.52%, respectively. Ls of SM, RM, and NM were 0.80, 0.82, and 0.76 times that of CK, respectively.

### 3.3 Carbon and nitrogen biogeochemical traits under different management modes

Management modes had a certain impact on the carbon and nitrogen biogeochemical traits (**Table 3**). TC ranged from 499.06 to 510.32 g·kg^-1^, and SM showed the highest TC. Although variations in TC were observed among management modes and CK, no significant differences were detected (**Table 3**).

**Table 3:** Carbon and nitrogen biogeochemical traits under different management modes.

Management modes	SM	RM	NM	CK
TC(g·kg^-1^)	510.32 ± 7.63a	507.74 ± 3.43a	499.06 ± 8.48a	505.66 ± 7.75a
TN(g·kg^-1^)	20.63 ± 1.01a	20.76 ± 0.73a	18.33 ± 1.02ab	17.36 ± 0.95b
C/N	24.82 ± 0.84b	24.51 ± 0.88b	27.35 ± 1.16ab	29.26 ± 1.21a
δ¹³C(‰)	-29.68 ± 0.75a	-29.11 ± 1.13a	-30.19 ± 0.59a	-30.21 ± 0.47a
δ¹^5^N(‰)	-4.40 ± 0.26a	-5.34 ± 0.10b	-4.68 ± 0.21ab	-5.25 ± 0.20b

TC, TN, C/N, δ¹³C and δ¹^5^N represent the total carbon, total nitrogen, the ratio of TC to TN, Carbon-13 isotope ratio, and Nitrogen-15 isotope ratio, respectively. Different lowercase letters within each row indicate significant differences among management modes (*p*< 0.05). Error bars indicate standard deviation (n = 3).

TN varied from 17.36 to 20.76 g·kg^-1^ and was higher under forest management treatments than under CK (**Table 3**). Significant differences in TN were found among SM, RM, and CK, with levels reaching 1.19 and 1.20 times that of CK (*p*< 0.05), whereas no significant differences were observed among the management modes.

In contrast to TN, C/N spanned from 24.51 to 29.26 and was lower under forest management treatments than under CK, with the lowest value observed under RM (**Table 3**). Significant differences in C/N were identified among SM, RM, and CK, with decreases of 15.20% and 16.23% relative to CK (*p*< 0.05), whereas no significant differences were observed among the management modes.

δ¹³C varied from -30.21 to -29.11‰ and was higher under forest management treatments than under CK, but these differences were not statistically significant (**Table 3**).

Unlike the δ¹³C, δ¹^5^N ranged from -5.34 to -4.40‰ (**Table 3**). Notably, SM significantly increased δ¹^5^N by 16.08% compared with CK (*p*< 0.05), while RM slightly decreased δ¹^5^N (by 1.81%, non-significant), resulting in a significant difference between SM and RM (*p*< 0.05). SM and NM increased δ¹^5^N by approximately 16.08% and 10.76%, respectively, relative to CK. Significant differences in δ¹^5^N were observed between SM and CK (*p*< 0.05), but not between RM and CK or between NM and CK. Moreover, SM also differed significantly from RM (*p*< 0.05) but not from NM.

### 3.4 Nitrogen use efficiency under different management modes

**Figure 3:**
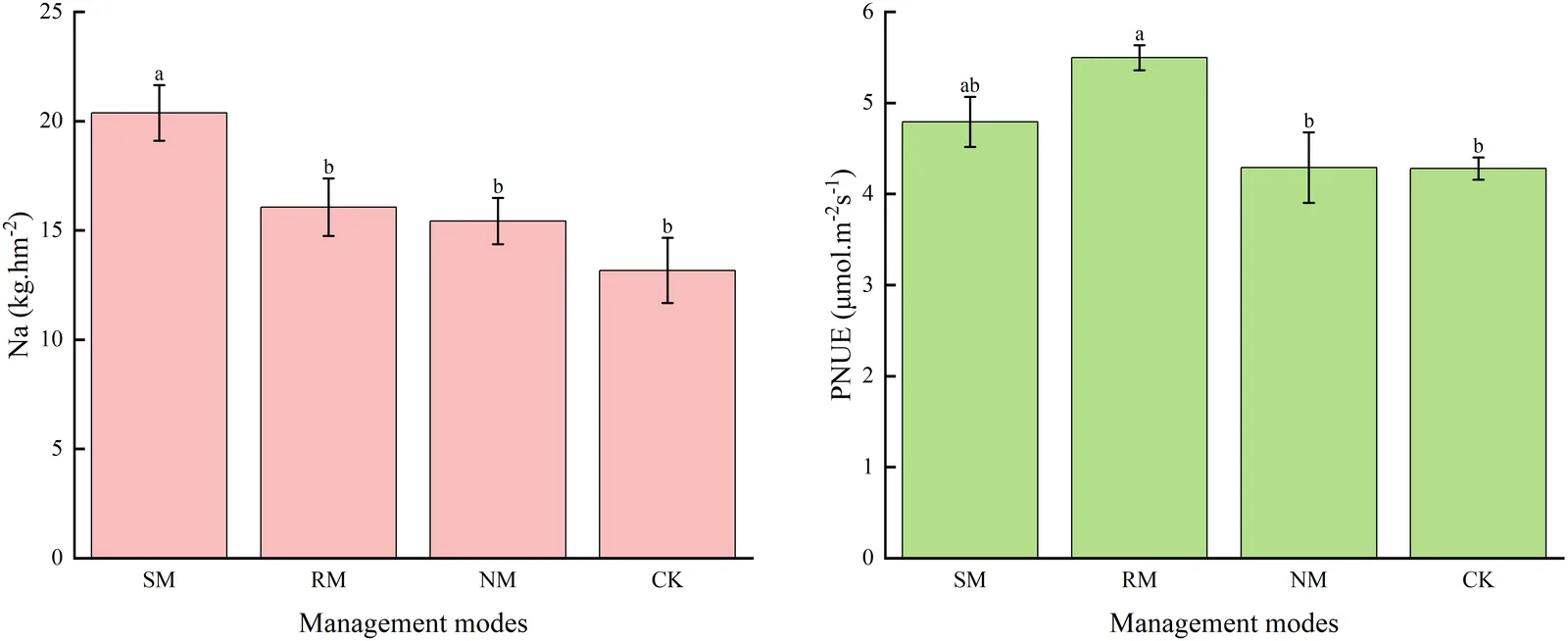
Na and PNUE under different management modes. Na and PNUE represent the nitrogen content in unit leaf and photosynthetic nitrogen use efficiency, respectively. Different lowercase letters indicate significant difference among management modes (*p*< 0.05). Error bars indicate standard deviation (n = 3).

**Figure 3** illustrates Na and PNUE under different management modes. Na varied from 13.17 to 20.38 kg·hm^-2^ and was higher under forest management treatments than under CK, with the highest value observed under SM. Significant differences in Na were displayed between SM and CK, with a level 1.55 times that of CK (*p*< 0.05), whereas no significant differences were observed among RM, NM, and the CK. Specifically, Na of SM was significantly different from that of RM and NM (*p*< 0.05), while no significant difference was found between RM and NM.

PNUE spanned from 4.28 to 5.50 μ mol·m^-^²·s^-^¹ and was higher under forest management treatments than under CK, with the highest value observed under RM. Significant differences in PNUE were displayed between RM and CK, with a level 1.28 times that of CK (*p*< 0.05), whereas no significant differences were observed among SM, NM, and CK. Additionally, RM showed a significant difference from NM in PNUE (*p*< 0.05), but not from SM. Specifically, PNUE of NM was almost equal to that of CK.

### 3.5 Relationships among morphological traits, gas exchange parameters, carbon and nitrogen biogeochemical traits, and nitrogen use efficiency

**Figure 4:**
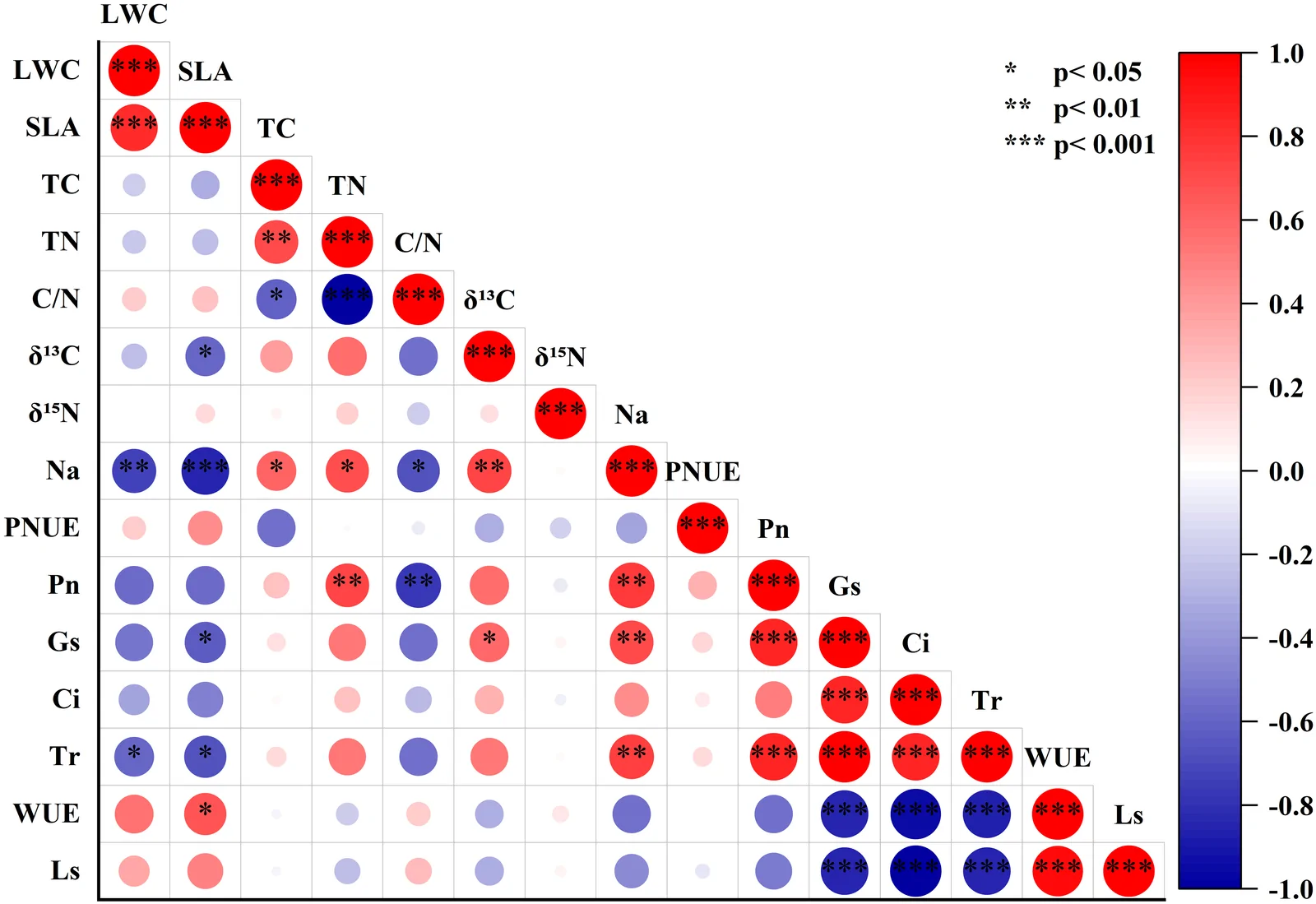
Correlation analysis among morphological traits, gas exchange parameters, carbon and nitrogen biogeochemical traits, and nitrogen use efficiency. *p< 0.05, **p< 0.01, ***p< 0.001. LWC and SLA represent the leaf water content and specific leaf area, respectively. TC, TN, C/N, δ¹³C and δ¹^5^N represent the total carbon, total nitrogen, the ratio of TC to TN, Carbon-13 isotope ratio, and Nitrogen-15 isotope ratio, respectively. Na and PNUE represent the nitrogen content in unit leaf and photosynthetic nitrogen use efficiency, respectively. Pn, Gs, Ci, Tr, WUE and Ls represent the net photosynthetic rate, stomatal conductance, intercellular CO_2_ concentration, transpiration rate, water use efficiency and stomatal limitation, respectively.

**Figure 4** presents the correlation analysis among morphological traits, gas exchange parameters, carbon and nitrogen biogeochemical traits, and nitrogen use efficiency.

SLA exhibited extremely significantly positive correlations with LWC but extremely significantly negative associations with Na (*p*< 0.001). Furthermore, SLA was significantly positively correlated with WUE but significantly negatively linked to δ¹³C, Gs, and Tr (*p*< 0.05). LWC was highly significantly negatively associated with Na (*p*< 0.01) and significantly negatively correlated with Tr (*p*< 0.05).

Pn, Gs, Ci, and Tr all displayed extremely significant positive correlations with each other (*p*< 0.001). In contrast, WUE showed extremely significant negative associations with Gs, Ci, and Tr (*p*< 0.001). Additionally, Pn was highly significantly positively correlated with TN and highly significantly negatively linked to C/N (*p*< 0.01). Gs was significantly positively associated with δ¹³C (*p*< 0.05). Specifically, Ls demonstrated extremely significant negative correlations with Gs, Ci, and Tr (*p*< 0.001) and extremely significant positive associations with WUE (*p*< 0.001).

Na was highly significantly positively correlated with Pn, Gs, Tr, and δ¹³C (*p*< 0.01) and also exhibited significant positive associations with TC and TN (*p*< 0.05). Notably, the positive correlation between Na and Pn (r = 0.82, p< 0.01) confirms nitrogen as a key driver of photosynthetic capacity. TC demonstrated highly significant positive correlations with TN (*p*< 0.01). Notably, δ¹^5^N and PNUE displayed weak or non-significant correlations with any of the other parameters.

## 4 Discussion

### 4.1 Effects of different management modes on morphological traits

LWC and SLA reflect plant water status and resource acquisition strategies. All management practices reduced the LWC and SLA of *P*. *tabulaeformis* needles, with SM showing the most significant decrease, suggesting that forest management—particularly SM—may induce more conservative water-use strategies in plants. This decline is likely attributable to the improved canopy light environment after management, which may increase transpiration. Furthermore, enhanced light availability could promote leaf sclerification by increasing mesophyll tissue density and leaf thickness, thereby improving tolerance to intense light and drought stress. Similar phenomena have been observed in related studies: SLA and LWC of *P*. *tabulaeformis* decrease under both full light and drought conditions (Yang X. et al., 2025; Fa et al., 2025).

Changes in leaf functional traits such as SLA can reflect plant adaptability to light and water resource availability (Jin et al., 2024). Low SLA is generally regarded as a “conservative” leaf economic spectrum strategy, indicating that plants tend to maintain longer leaf lifespans and reduce resource investment (Wright et al., 2004). Lower SLA implies greater leaf thickness and denser tissues, which helps improve water use efficiency and stress resistance in resource-limited environments (Jin et al., 2024). This morphological adjustment—increasing investment in leaf structural defenses and persistence—may represent a structural response to post-management microenvironmental changes (e.g., increased light intensity and intensified competition), thereby enhancing water retention capacity or reducing transpirational losses. This finding is consistent with numerous studies on environmental stress (e.g., water stress) that cause plants to reduce SLA to improve drought resistance (Di et al., 2009). The increased forest light transmittance resulting from management in this study may have produced physiological effects analogous to a “mild drought” stress, driving leaf morphology toward a conservative type. This result also supports the conclusion that plants respond to environmental changes by adjusting their biophysical characteristics (Yang Q. et al., 2025).

The significant decrease in LWC under the management regimes reflects increased transpiration and water loss due to enhanced light radiation after management (Cachinero-Vivar et al., 2024). Plants optimize water balance by reducing leaf moisture content. In this study, although LWC decreased, both Gs and Tr significantly increased under SM, suggesting that plants effectively compensate for reduced water-holding capacity through enhanced water transport or stomatal regulation, thereby maintaining high photosynthetic carbon fixation.

Overall, the observed responses indicate that plants adopt a resource conservation strategy in post-management environments characterized by increased light exposure and associated transpiration pressure: investing in constructing thicker, lower-water-content leaves to enhance water retention capacity and structural defense. This finding aligns with the classic “trade-off” theory in plant ecology. Importantly, the enhanced light conditions induced by management in this study did not elicit the typical “high-investment, high-return” leaf morphology but instead triggered adaptation mechanisms similar to drought stress, prompting *P*. *tabulaeformis* to prioritize water security. This response pattern is analogous to findings from studies on pine species conducted in arid or semi-arid regions, which suggest that water availability is a key factor driving leaf morphological changes in coniferous tree species. This morphological shift towards water conservation, however, does not necessarily translate into reduced gas exchange, as shown in the next section.

### 4.2 Effects of different management modes on gas exchange parameters

Photosynthesis is central to forest carbon fixation, directly dictating tree growth rate and productivity. In this study, all management modes (SM, RM, and NM) significantly increased the Pn of *P*. *tabulaeformis* relative to CK, with RM yielding the largest enhancement. This finding suggests that management interventions improve light availability and reduce competitive intensity within the stand, thereby boosting leaf photosynthetic carbon assimilation. Our results are in strong agreement with previous studies showing that thinning or gap disturbances that induce light penetration through the canopy enhance photosynthetic performance (Cachinero-Vivar et al., 2024). Empirical evidence has also shown that thinning notably elevates Pn in larch plantations (Wu et al., 2025) and improves photosynthetic capacity in *Pinus banksiana* and *Picea mariana* (Goudiaby et al., 2011), as well as in *P*. *tabulaeformis* (Zhao and Bao, 2018). RM likely creates a more heterogeneous stand structure that provides a superior light microenvironment, maximally alleviating light limitation – a conclusion that echoes a study reporting the highest photosynthetic capacity in young *P*. *tabulaeformis* under moderate shading (Wang et al., 2016).

Both Gs and Tr followed trends similar to those of Pn, indicating that management reduces stomatal restriction and enhances CO_2_ uptake and water loss. Studies have shown that when disturbed by thinning, trees can activate physiological responses at the leaf level to high light and water stresses, such as increased Gs and stomatal index (Moretti et al., 2024). Among the modes, only SM significantly promoted Gs and Tr, whereas RM and NM showed non-significant increases in both parameters. This discrepancy likely arises from mode-specific alterations to canopy architecture. By systematically optimizing stand spatial structure, SM may increase canopy openness to a greater extent, which substantially enhances photosynthetically active radiation within the canopy, thereby effectively activating stomatal opening. In contrast, although RM and NM also raised the mean light intensity inside the stand, the resulting increase in canopy openness is comparatively smaller, making it difficult to generate sustained, stable high-light zones. Consequently, their capacity to promote stomatal conductance and transpiration rate was limited. Research confirms that gap-cutting at a fine scale can enhance stand structural heterogeneity and provide ample light and soil moisture to initiate regeneration (Tinya et al., 2025). Similar photosynthetic enhancements in response to thinning have been reported for other coniferous species. For instance, thinning has been shown to significantly increase light-saturated net photosynthetic rates in Scots pine (*Pinus sylvestris*) (Portsmuth and Niinemets, 2007) and to enhance photosynthetic capacity in Norway spruce (*Picea abies*) (Gspaltl et al., 2013). These findings suggest that conifers generally share a common physiological strategy—increasing stomatal conductance and photosynthetic capacity when light availability improves—though the magnitude of response may vary with species’ shade tolerance and water-use strategies.

Ci tended to increase under management, whereas WUE and Ls decreased. This pattern implies that the post-management photosynthetic improvement is likely associated with increased CO_2_ supply via stomatal opening (Soto et al., 2024), alongside a possible enhancement in mesophyll conductance or Rubisco carboxylation capacity—mechanisms that cannot be resolved with our current dataset. Nonetheless, the observed increase in both Pn and Ci suggests that stomatal limitation was alleviated under management, and that biochemical capacity may also have been upregulated. Future A-Ci curve measurements are needed to disentangle these contributions. Consequently, WUE declined along with the higher transpiration rates. Such a “stomatal-driven photosynthetic enhancement” – a trade-off distinct from the conventional view that high Pn often accompanies high WUE – is widely documented in plant physiology. It mirrors mechanisms reported for *Pistacia chinensis* (Huang et al., 2023) and aligns with response patterns of *P*. *tabulaeformis* along environmental gradients (Di et al., 2012; Yang X. et al., 2025). Furthermore, the simultaneous increase in both Ci and Pn suggests that management may also enhance leaf biochemical capacity, including Rubisco activity and ribulose-1, 5-bisphosphate (RuBP) regeneration (Namachivayam et al., 2025; Suzuki et al., 2021).

In summary, SM significantly promotes photosynthetic physiology by raising Pn, Gs, and Tr. However, its effects on Ci, instantaneous WUE, and Ls were not statistically significant, possibly because the increase in Pn under SM was driven more by elevated Gs than by changes in Ci, and because instantaneous WUE reflects a short-term trade-off that may not capture longer-term carbon-water balance. This hints at the existence of an optimal light environment for improving growth and yield (Li et al., 2026). From a management perspective, while SM maximizes photosynthetic gains, its potential compromise on water use efficiency suggests that a hybrid approach could be tested in future trials to balance carbon gain and water conservation, especially in water-limited regions (Khan et al., 2025). Additional measurements, such as light and CO_2_ response curves, chlorophyll fluorescence parameters (e.g., Fv/Fm, NPQ) and δ¹³C analysis for long-term WUE, are needed to disentangle stomatal from biochemical limitations and to determine the integrated carbon-water economy (Bhattarai et al., 2025).

### 4.3 Effects of different management modes on carbon and nitrogen biogeochemical traits and nitrogen use efficiency

Both SM and RM regimes significantly increased TN and reduced the C/N. These results indicate that management practices, by improving the within-stand light environment and alleviating competitive pressure, promoted soil nitrogen uptake and subsequent nitrogen accumulation in the needles of *P*. *tabulaeformis*. Such changes may be attributable to accelerated litter decomposition, enhanced soil nitrogen mineralization, or increased root growth vigor following management (Qu and Hai, 2025). TC did not differ significantly among treatments, consistent with its role as a stable structural element. Correlation analysis further revealed that Pn was significantly positively correlated with TN and significantly negatively correlated with C/N, confirming the central role of leaf nitrogen accumulation in driving photosynthetic capacity (Gao et al., 2014). From the perspective of carbon-nitrogen metabolism, the decline in C/N reflects a shift in resource allocation toward nitrogen acquisition and the construction of photosynthetic machinery. This synergistic adjustment of carbon and nitrogen metabolism represents an adaptive response of *P*. *tabulaeformis* to the improved habitat conditions created by management (Han and Chiba, 2009). A global-scale meta-analysis also demonstrated that thinning can significantly reduce C/N, a finding that aligns well with the results of the present study (Fan et al., 2025).

As an integrated indicator of long-term WUE, δ¹³C showed no statistically significant differences among management regimes but exhibited a tendency to be higher than in the control. This pattern contrasts with the decrease in instantaneous WUE. This apparent discrepancy may reflect differences in the water-use strategy of *P*. *tabulaeformis* across temporal scales: the improved light environment after management promotes more open stomata on a daily scale, leading to lower instantaneous WUE, whereas enhanced long-term carbon gain might compensate for increased water loss, thereby maintaining δ¹³C relatively stable or even slightly elevated (Dietrich et al., 2025). δ¹^5^N values diverged among management regimes: they increased under SM and NM, but decreased slightly (non-significantly) under RM. This divergence may be associated with management-induced alterations in the nitrogen sources available to the trees, involving shifts in mycorrhizal symbiosis efficiency, soil organic nitrogen mineralization rate, or the proportion of inorganic nitrogen taken up by plants. Notably, the significant increase in δ¹^5^N under SM is likely related to enhanced soil organic nitrogen mineralization and an increased uptake of inorganic nitrogen from the soil following management (Qu and Hai, 2025).

Nitrogen is a key element limiting forest growth, and its cycling is regulated by plant community structure and environmental factors (Fang et al., 2024). PNUE is a critical physiological metric that quantifies how efficiently a plant invests leaf nitrogen into photosynthetic carbon assimilation. It is a core component of the “leaf economics spectrum” and is linked to traits such as leaf nutrient content, photosynthesis, respiration, leaf lifespan, and dry-mass investment (Wright et al., 2004). Our study revealed divergent trends in Na and PNUE under different management modes.

SM significantly increased Na, but did not significantly enhance PNUE, suggesting indicating that under SM, the additional nitrogen taken up is not being translated into proportional increases in photosynthetic efficiency. This pattern is consistent with —though not definitive proof of—a strategy in which nitrogen may be preferentially allocated to storage or defensive pools rather than to the photosynthetic apparatus. Direct measurements of nitrogen partitioning (e.g., Rubisco content, chlorophyll concentration, storage proteins) are required to validate this interpretation. The significant positive correlation between Na and Pn supports the notion that nitrogen is a limiting factor, but the lack of a corresponding increase in PNUE suggests diminishing marginal returns. In conifers such as Douglas-fir, leaf Na also shows a negative relationship with PNUE, indicating that excessive nitrogen accumulation does not necessarily translate into higher photosynthetic efficiency (Ripullone et al., 2003).

In contrast, RM significantly increased PNUE without a marked increase in Na, suggesting that RM adopts a “nitrogen utilization” strategy. By maintaining a relatively low and stable Na level, this regime optimizes the allocation of nitrogen among photosynthetic components (e.g., increasing the catalytic efficiency of Rubisco or the proportion of nitrogen invested in chlorophyll), thereby achieving higher PNUE and greater nitrogen use economy. This conclusion is consistent with the established understanding that stand structural heterogeneity (e.g., mixed species or structural adjustments) can alter nitrogen uptake and utilization patterns among tree species (Wu et al., 2019). The observed increase in PNUE is not only a consequence of higher Pn but is also closely related to the elevation of TN. Such an efficient nitrogen use strategy is of great significance for enhancing the long-term competitive ability of trees, especially in nutrient-limited ecosystems. In this context, the RM regime demonstrates an effective approach to reshaping intrinsic plant physiological mechanisms through external intervention.

### 4.4 Coordination and trade-offs among leaf functional traits and photosynthetic characteristics

Correlation analyses revealed complex coordination and trade-off relationships among leaf functional traits and photosynthetic characteristics in *P*. *tabulaeformis*. The positive correlation between SLA and LWC and the negative correlation between SLA and Na (**Figure 4**) further support the management-induced “hardening shift” toward thicker, denser leaves, consistent with an “investment-return” trade-off (Khan et al., 2022).

Notably, SLA was positively correlated with WUE but negatively correlated with δ¹³C, Gs, and Tr (**Figure 4**). This relationship suggests that under our experimental conditions, lower SLA is not favorable for improving WUE. In our study, this finding is partially inconsistent with studies reporting that “leaves with low SLA have higher mesophyll tissue density, thereby reducing water loss and enhancing WUE” (Petrík et al., 2024; Rawat et al., 2021). Potential explanations include: (1) management practices improved light, water, and nutrient conditions within the stand; leaves with low SLA, owing to their higher nitrogen content, exhibited enhanced photosynthetic capacity, which partially offset water consumption; (2) as a coniferous species, *P*. *tabulaeformis* has a relatively narrow range of SLA variation, and WUE is more directly influenced by stomatal regulation (strong negative correlation between WUE and Gs). Thus, in this study, SLA likely affects WUE indirectly through nitrogen allocation rather than via the mesophyll tissue density pathway. Recent studies have indicated that the carbon assimilation–nitrogen relationship in conifers differs fundamentally from that in broadleaved species (Ripullone et al., 2003), offering an interspecific perspective to explain the distinct SLA–WUE relationship observed here.

Pn, Ci, Gs, and Tr were all highly significantly positively correlated, fully reflecting tight couplings between stomatal behavior and photosynthetic carbon assimilation during gas exchange. This coupling was enhanced under all three forest management regimes (SM, RM, NM) (**Figure 2**), indicating that management practices, by improving the light environment within the stand, simultaneously activated stomatal opening and the operation of the photosynthetic electron transport chain. Similar carbon assimilation–stomatal coupling has been observed in other coniferous species such as *Pinus koraiensis*, where nitrogen addition significantly promoted both Pn and Gs (Tian et al., 2025).

WUE was highly significantly negatively correlated with Pn, Ci, Gs, and Tr, highlighting the fundamental trade-off between carbon gain and water consumption (Di et al., 2012). This carbon–water balance trade-off can be quantified using δ¹³C stable isotopes (Lawson et al., 2025). Studies have shown that intrinsic WUE (iWUE) in evergreen conifers has declined in recent years, and the carbon–water balance responses differ significantly among plant functional types (Malcomb et al., 2025). These general relationships suggest that management practices integrally affect the ecological functions of *P*. *tabulaeformis* by altering a suite of interconnected traits (Quan and Wang, 2015).

From a nutrient regulation perspective, the highly significant positive correlation between Pn and TN and the highly significant negative correlation between Pn and C/N constitute the core physiological chain through which management practices drive photosynthetic efficiency: management practices, by promoting foliar nitrogen accumulation, reduce the leaf C/N, thereby providing sufficient nitrogen substrate for the construction of photosynthetic apparatus (Reich et al., 1999). Similarly, a study on *Larix gmelinii* found that nitrogen addition significantly increased maximum net photosynthetic rate, maximum carboxylation rate, and maximum electron transport rate (Cai et al., 2025), further supporting the general role of nitrogen in regulating photosynthetic capacity. Furthermore, Na was highly significantly positively correlated with Pn, Gs, Tr, and δ¹³C, and significantly positively correlated with TC and TN (**Figure 4**); however, Na was significantly negatively correlated with LWC and SLA. This seemingly contradictory pattern actually reveals an important resource allocation strategy in *P*. *tabulaeformis* needles under management: as nitrogen investment in needles increases (higher Na), needles tend to become thicker, with lower SLA and lower LWC. This structural “hardening” helps maintain higher Pn and Gs under high-light conditions, thereby enhancing carbon assimilation capacity. Thus, Na drives a functional shift from “high investment–low efficiency” to “high investment–high return”, reflecting the positive effect of nitrogen investment on photosynthetic capacity enhancement. This interpretation, together with the Pn–TN positive correlation and the Pn–C/N negative correlation, forms a complete nutrient–photosynthesis synergy chain.

Of note, δ¹^5^N increased significantly under SM but showed weak or non-significant correlations with most physiological parameters in the correlation analysis (**Table 3**, **Figure 4**). The weak correlation between δ¹^5^N and PNUE may reflect time-lags in nitrogen isotope incorporation or the influence of mycorrhizal mediation on nitrogen sources. Additionally, changes in δ¹^5^N may primarily indicate shifts in soil nitrogen source pools (e.g., nitrification versus organic N mineralization) rather than directly affecting instantaneous nitrogen-use efficiency (Dietrich et al., 2025). Therefore, while SM altered the nitrogen cycling pathway (as reflected by δ¹^5^N enrichment), this change did not immediately translate into enhanced photosynthetic nitrogen-use efficiency, possibly due to time lags in leaf protein turnover or reallocation of nitrogen to storage compounds. The stable isotope δ¹^5^N can serve as an integrated indicator of soil nitrogen cycling and nitrogen source composition; forest management can influence the distribution pattern of δ¹^5^N within ecosystems by altering canopy structure and soil microenvironments (Ibañez et al., 2025). Unlike δ¹^5^N, PNUE, although significantly higher under RM than under CK (**Figure 3**), did not show strong associations with other traits in the correlation network (**Figure 4**). This may indicate that PNUE is comprehensively regulated by multiple factors (e.g., nitrogen allocation strategy, mesophyll conductance) and responds relatively independently. Site conditions and stand composition significantly regulate PNUE, and the responses of PNUE to management practices differ among branches of different ages (Kharel et al., 2025).

Finally, the correlation network in this study clearly reveals the complex trade-off relationships among leaf functional traits in *P*. *tabulaeformis*. Among these, the “photosynthesis–stomatal conductance–transpiration synergy” and the “carbon gain versus water consumption trade-off” are the most prominent patterns in **Figure 4**. Considering the effects of different management regimes: SM significantly increased Na and δ¹^5^N, as well as Pn, Gs, and Tr (**Table 3**, **Figures 2** and **3**), suggesting that SM may enhance photosynthetic capacity by promoting nitrogen uptake and accumulation, although direct biochemical evidence is needed to confirm this interpretation. RM, although not significantly increasing Na, exhibited the highest PNUE (**Figure 3**) and the highest Pn (not significantly different from SM) (**Figure 2**), suggesting that RM focuses more on increasing photosynthetic output per unit nitrogen. NM showed intermediate effects. Collectively, these relationships reflect the ecophysiological strategies adopted by P. *tabulaeformis* to adapt to environmental changes under different management regimes, providing important data support for understanding how plants coordinate resource utilization and respond to management disturbances.

### 4.5 Limitations

Several interrelated methodological constraints should be considered when interpreting our findings. First, our measurements were conducted during a single growing season at one site with limited replication (n = 3 per treatment), which restricts both the temporal and spatial generalizability of our conclusions. Although statistically significant differences were detected, the absence of inter-annual replication leaves the temporal stability of these physiological responses unknown. Second, our analysis was restricted to current-year needles. Given that *P. tabulaeformis* is an evergreen conifer with a needle lifespan of 3–5 years, older needle cohorts may exhibit distinct photosynthetic capacities, nitrogen allocation patterns, and water-use strategies (Wang Y. et al., 2022). Our PNUE estimates based solely on current-year needles may therefore overestimate whole-plant nitrogen-use efficiency, as older needles typically undergo nitrogen resorption. Third, the observed photosynthetic enhancement under management was inferred from instantaneous gas exchange measurements without concomitant biochemical parameterization (e.g., maximum carboxylation rate, mesophyll conductance, or nitrogen partitioning between Rubisco and chlorophyll). Consequently, the relative contributions of stomatal versus non-stomatal factors to the increased *Pn* remain unresolved. Finally, the interpretation of foliar δ¹^5^N shifts is limited by the absence of concurrent soil nitrogen dynamics data, which precludes distinguishing between changes in N source versus N cycling pathways.

Notwithstanding these constraints, the consistent trait differentiation across management regimes provides a clear mechanistic basis for future investigations. We frame our findings as preliminary evidence that management-induced structural changes exert measurable short-term effects on leaf carbon, water, and nitrogen economies. Multi-year, multi-site studies, coupled with biochemical analyses (A-Ci curves, nitrogen partitioning coefficients) and soil dynamic assessments, are essential to validate the sustainability and generalizability of these observed patterns across broader environmental contexts and temporal scales. Future research should also integrate long-term stand growth (e.g., biomass increment, timber yield) monitoring to assess whether leaf-level physiological gains translate into stand-level productivity improvements.

## 5 Conclusions

This study revealed that different forest management modes (SM, RM, and NM) significantly altered needle morphology, photosynthetic physiology, and nitrogen use efficiency in *P*. *tabulaeformis* plantations within a single growing season.

Our findings can be summarized in three key observations. First, a coordinated shift towards a conservative strategy: all management modes significantly reduced LWC, with SM showing the largest decrease (8.44%), and SM also significantly reduced SLA by 27.3%, indicating management-induced needle sclerification. Coupled with maintained or slightly elevated δ¹³C and increased Na under SM, these changes reflect a multi-trait conservative syndrome—characterized by enhanced structural resistance, improved long-term water conservation, and increased nitrogen investment per unit area—representing an integrated adaptive response to management-altered microenvironmental conditions. Second, a photosynthetic-water consumption trade-off: management substantially improved photosynthetic performance at the cost of water loss. SM, RM, and NM increased Pn by 57%, 59%, and 33%, respectively, while all treatments decreased WUE, revealing a fundamental trade-off between carbon gain and water consumption. Third, contrasting nitrogen-use patterns: SM significantly increased Na and elevated δ¹^5^N, a pattern consistent with enhanced nitrogen uptake and accumulation, whereas RM significantly increased PNUE without markedly increasing Na, a pattern consistent with more efficient nitrogen utilization. However, these patterns are based on current-year needles and inferred from bulk nitrogen content and PNUE; direct biochemical evidence and multi-age cohort analyses are required to substantiate these interpretations.

Overall, the synergistic and trade-off networks among traits support RM may offer a favorable balance between carbon assimilation and nitrogen economy. Among the three modes, RM achieved the highest Pn (+59%, comparable to SM) and the highest PNUE (+28%) while avoiding excessive nitrogen accumulation. Therefore, RM appears to be a promising management approach *P. tabulaeformis* plantations to simultaneously enhance carbon fixation and maintain high nitrogen-use efficiency. While our findings provide robust evidence of immediate physiological responses to management within a single growing season, their temporal stability and broader generalizability require confirmation through longer-term, multi-year observations.

## Data Availability

The original contributions presented in the study are included in the article/supplementary material. Further inquiries can be directed to the corresponding authors.
